# Decompressive craniectomy for traumatic intracranial hypertension: application in children

**DOI:** 10.1007/s00381-017-3534-7

**Published:** 2017-09-06

**Authors:** Adam M. H. Young, Angelos G. Kolias, Peter J. Hutchinson

**Affiliations:** 1 Division of Neurosurgery, Department of Clinical Neurosciences, Addenbrooke’s Hospital & University of Cambridge, Cambridge, UK; 2 Department of Academic Neurosurgery, Addenbrooke’s Hospital, University of Cambridge, Cambridge, UK

**Keywords:** Brain, Injury, Craniectomy, Decompression, Paediatric

## Abstract

Traumatic brain injury remains prevalent in children, particularly within the adolescent age group. In severe injury, the priority of treatment is to stabilise the patient initially and prevent the evolution of brain swelling and secondary ischaemia using tiers of medical therapy. The final stage of intervention for such patients is a decompressive craniectomy. Here in, we identify the current evidence for performing decompressive crainectomy in children including the results from the RESCUEicp study.

## Introduction

The greatest clinical challenge after a traumatic brain injury is to minimise secondary injury to the brain [1]. The complex, dynamic changes that occur in the brain’s physiology and chemistry can lead to progressive swelling in the brain. This results in reduced blood flow, limited oxygenation and ultimately poor outcome [2–5]. Advances in multi-modality monitoring parameters and intensive care management have allowed for an improvement in understanding of the optimal physiological targets in adults after a traumatic brain injury [6]. This has helped to guide both medical and surgical intervention in these patients with improving outcomes. Nevertheless, these benefits have been slower to translate into paediatric care after a traumatic brain injury (TBI). As such, the optimal strategies of both medical and surgical intervention remain widely debated [7].

Intracranial hypertension can occur in up to 65% of patients after a severe paediatric TBI, moreover, raised intracranial pressure (ICP) accounts for over half of all TBI-related deaths [8, 9]. The total time that ICP is elevated to greater than 20 mmHg correlates strongly with outcome [10]. Therapeutic interventions for reducing ICP include reducing the intracranial contents by removing CSF, reducing blood volume, or brain volume, by reducing cerebral metabolic demands or by increasing cranial volume by decompressive craniectomy (DC).

**Fig. 1:**
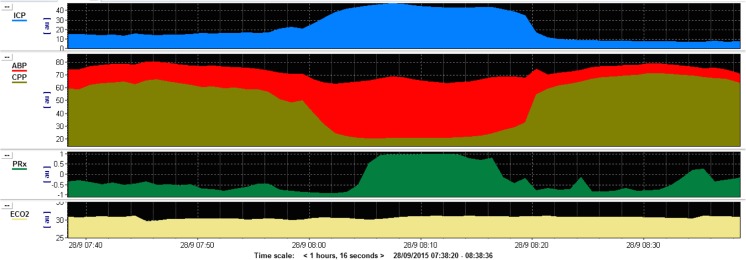
This figure shows an example monitoring trace of a patient with intracranial hypertension as a result of a traumatic brain injury. The trace demonstrates a sustained plateau of intracranial pressure (*blue*) that lasts for around 20 min. This is associated with a reduced cerebral perfusion pressure (*red*), and as a result, the brain’s cerebral autoregulation is non-compliant (*green*). These are key features that can occur with severe traumatic brain injury and if recurrent would demonstrate potential benefit in undertaking a decompressive craniectomy

Cranial decompression is regarded as the final stage of intracranial hypertension management with its efficacy debated widely in the context of both paediatric and adult TBI [11–22]. The procedure can greatly improve brain compliance and improve compensatory reserve (Fig. 1) [23, 24]. As a consequence, improved cerebral blood flow and cerebral perfusion pressure (CPP) will augment brain tissue oxygenation (PbtO2).

The classical surgical options include unilateral hemicraniectomies [13, 14, 18, 25] and bifrontal hemicraniectomies [16, 22, 26]. However, less commonly bilateral hemicraniectomies [14, 18], circumferential craniectomies [27] and bilateral temporal craniotomies [15, 19] have been implemented all of which involve the excision of large sections of the skull +/− duraplasty.

## Indications

**Fig. 2:**
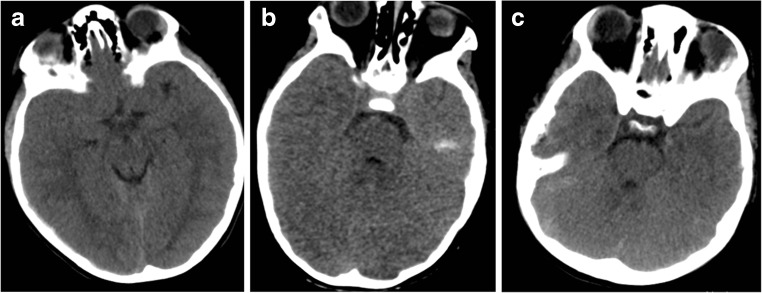
Representative image of paediatric patients with raised intracranial pressure. **a** Fourteen-year-old patient with acute subdural haematoma (ASDH), opening ICP 32 mmHg. **b** Seven-year-old patient with diffuse axonal injury (DAI), opening ICP 38 mmHg. **c** Twelve-year-old patient with ASDH and DAI opening pressure 35 mmHg. All patients demonstrate open basal cisterns despite pathologically raised ICP

The indications for a DC in a child are not uniformly agreed on. Younger patients generally have a better outcome, nevertheless, given the implications of the procedure caution should be encouraged with patient selection [12, 21, 28]. Features such as brainstem injury and central herniation would generally exclude a patient from such a procedure because of the pre-disposition to poor outcome [29, 30]. The post-resuscitation GCS is perhaps the most accurate assessment [31, 32]. The presence of radiological features is often helpful in determining the necessity to decompress. Midline shift of the brain on computed tomography (CT) is highly prognostic in children with the degree of shift being inversely related to outcome [33]. Interestingly, in adults, where an absence or compression of the basal cisterns is predictive of poor outcome [34], in the paediatric population, patent basal cisterns do not exclude high ICP [35] and extra caution should be observed (Fig. 2). Recent evidence suggests that optic nerve sheath diameter (ONSD) is particularly accurate in predicting current ICP in paediatric patients [36]. Regardless, the most accurate assessment one can make is that of invasive intracranial pressure and cerebral perfusion pressure [10, 37, 38].

Early decompression was thought to be related to a better outcome. That said, premature intervention may be unnecessary and must be balanced against the knowledge that a delayed intervention can result in irreversible injury and increased risk of neurological damage. However, the DECRA trial, which was published in 2011, recruited 155 adult patients with severe diffuse TBI and moderate intracranial hypertension. Patients were randomised within the first 72 h following TBI if their ICP exceeded 20 mmHg for >15 min—continuously or intermittently within a 1-h period, and if they did not respond to optimised first-tier ICP-lowering interventions. The two arms of the trial were bifrontal DC and standard medical management or standard medical management alone. Patients in the surgical arm had a higher rate of unfavourable outcomes (70 versus 51%; OR 2.21; 95% CI 1.14–4.26; *p* = 0.02) [12]. DC when used appropriately can be lifesaving but may come at the expense of severe neurological impairment.

A stepwise approach to care should be implemented without delay. Only when maximal medical therapy has been exhausted should DC be considered. The initial management of patients should encompass elevation of the head to 30 degrees, sedatives both with and without paralytics, adjustment of ventilatory settings to maintain PaCO2 at 30–35 mmHg, cooling to at least normothermia with a number of centres arguing that hypothermia is also beneficial [39], maintenance of hyperosmolar euvolaemia, correcting hyperglycaemia, supporting CPP with vasopressors and CSF diversion.

Specifically in children, evidence to support thresholds for ICP and CPP is limited. The general consensus is that an ICP greater than 20 mmHg and a CPP below 50 mmHg are consistent with poor outcome [10, 37, 38]. ICP in the low twenties is generally accepted so long that CPP and PbtO2 are well maintained. Further work is required to provide age-specific or even weight-specific pressure thresholds.

## Current practice in paediatrics

Generally, the evidence describing the benefits of DC in paediatric TBI patients is limited. Eight class III studies were reviewed for the publication of paediatric TBI management guidelines [40]. The studies vary in terms of the inclusion criteria and also in the intervention performed.

### Decompressive craniectomy for control of ICP

A misconception with the implementation of DC is that it is used to reduce the ICP from pre-operative values. Whilst this often results, this is not the primary aim. Rather, the aim is to lower medically intractable ICP such that minimal therapeutic intervention is required to optimise post-operative pressures [40]. Similar to adults, the emphasis of paediatric decompression is with early intervention. [41] describes a complete survival rate of seven patients who presented “massive” bilateral or unilateral swelling, compressed supratentorial swelling and perimesencephalic cisterns. Patients were treated with fronto-temporal DC all within 70 min from ictus. The initial ICP exceeded 45 mmHg in all patients, with six of seven achieving ICP of <20 mmHg post-operatively. The final patient was controlled well with medical therapy.

In contrast, [42] favoured the presence of prolonged ICP plateaus to determine which patients would benefit from surgery. Children who had a sustained ICP of over 20 mmHg for over 30 min were treated with a unilateral DC with duraplasty. In five of six patients, ICP was appropriately controlled in the sixth patient uncontrolled ICP promoted a return to surgery for a contralateral procedure.

[43] described a case series of 23 patients with a TBI under 20 years who underwent DC for initial mass lesion requiring evacuation or elevated ICP that was refractory to medical management. Bifrontal/biparietal craniectomies with duraplasty and sectioning of the falx were the most common performed within the series. Unilateral DC was utilised in the presence of a mass lesion or unilateral swelling. Ten patients underwent an early DC with 11 having a later DC. The mean ICP was significantly reduced from 30 to 18 mmHg post-operatively. All but two patients had medically managed ICP after decompression. [44] observed similar findings in patients who had either mass lesions removed or primary brain swelling decompressed, selected for having an ICP >25 mmHg or evidence of herniation on CT. Of those that underwent decompression, only one required management of sustained ICP elevations post-operatively. [45] described using bifrontal/biparietal craniotomies with duraplasty to control ICP in four of the five patients in his study.

Finally, [46] described similar findings in a case series of 23 patients under 2 years of age who underwent surgery as a result of non-accidental injury. Decision to decompress was based purely on the ICP. Of the nine patients who were decompressed, DC lowered the mean ICP from 54.9 to 11.9 mmHg. Surgery in all patients was performed within 24 h of injury.

### Decompressive craniotomy for improving outcome

The impact of decompression on the outcome of children with intracranial hypertension is unclear. In one prospective study, [19] reported a favourable trend of the effect of decompression on ICP and outcome in a small pilot clinical trial of decompressive craniectomy in 27 children (13 of whom were treated with decompression). In addition, several case series have been published that collectively describe very early application of either unilateral or bilateral decompression in 11 paediatric cases [7, 42]. In some of these cases, decompression was the therapeutic choice prior to maximal medical management. Specifically, [7] describes Glasgow Outcome Scores of 4–5 at 40-month follow-up with [42] citing similar results at 6 months in seven of 11 patients.

A retrospective study on 23 patients who underwent decompression for diffuse axonal injury or contusions reported survival in 16 patients, 13 with favourable outcome [43]. Mannitol, hypertonic saline, neuromuscular blockade and controlled ventilation (targeting a PaCO2 of ~35 mmHg) were used as the medical management strategies, and surgery was undertaken if ICP remained >20 mmHg on this regimen.

Decompression has been reported to have a negative impact in two studies [44, 47]. Managed using the Lund approach five of eight patients displayed significant neurological deterioration at the time of surgery. Nevertheless, all but one survived with three patients having a full neurological recovery.

In one of the larger studies, 51 patients underwent decompressive craniectomy, mainly for evacuation of a mass lesion [44]. Five cases were performed for the refractory ICP >25 mmHg and the sixth for clinical herniation. Five of the six patients died.

### RESCUEicp

This international, multicentre, parallel-group, superiority, randomised trial assessed the comparative effectiveness of craniectomy versus advanced medical management (with the option of barbiturates), for patients with severe and refractory intracranial hypertension. The trial included patients aged 10 to 65 with a TBI and raised intracranial pressure (>25 mmHg for 1 to 12 h, despite stage 1 and 2 measures). In total, 408 patients were randomised, of whom 56 were ≤18 years and 16 patients were ≤16. The primary analysis showed a significant between-group difference in the extended Glasgow Outcome Scale (GOS-E) distribution and a substantial reduction in mortality with surgery. The pre-specified sensitivity analysis dichotomized at upper severe disability (independent at home) or better was significant at 12 months (i.e. 45.4% of the patients in the surgical group were at least independent at home, as compared with 32.4% of patients in the medical group; *p* = 0.01). Furthermore, we estimated that treating 100 patients with craniectomy as opposed to medical treatment would result in 22 more survivors of whom, at 12 months, almost 60% will be at least independent at home.

## Complications

Frequently after decompression, the refractory swelling that results from a hyperaemic state can cause strangulation and herniation, particularly if the craniectomy is of insufficient size.

The most frequent complication that arises from decompressive surgery is disruption of CSF dynamics. In adults, hydrocephalus occurs between 14 and 29% of patients and hygromas in 26% [48–50]. Other complications include cerebral ischemia, infection, wound dehiscence, seizures, syndrome of the trephined and secondary brain injury as a result of an unprotected brain.

Hydrocephalus as a result of the trauma must be distinguished from that as a result of surgery. The resulting increase in pressure can promote CSF leak from the skin and predispose to infection. Options for treating hydrocephalus include external ventricular drainage, ventriculoperitoneal shunting or lumbar drainage if the cisterns allow. Frequently when the bone is replaced, hydrocephalus *ex vacuo* may resolve with only a subcohort of patients requiring a shunt after cranioplasty.

Hygromas usually occur on the ipsilateral side following surgery [48, 49]. Although most resolve spontaneously without intervention on occasion, they can require drainage or CSF diversion [48, 49], in children externalisation of drains is avoided where possible. Percutaneous drainage of epidural CSF collections may also be considered. On the whole replacement of the bone flap will usually improve CSF dynamics.

The extensive scalp incision and bony resection involved in craniectomy combined with lengthy intensive care unit stays and multiple invasive procedures all combined to increase the risk of infection in patients’ post-decompression. In addition to the 3–6% of infection, wound dehiscence is a major concern. Particularly, common in younger children due to the depth of the scalp, the tension expressed by the expanding brain can frequently cause the wound to open.

Seizures occur in both the acute and chronic phases of treatment with persistent seizures requiring medical intervention occurring in up to 5% of patients [48]. The reduction of Bovie cautery when replacing the bone flap has been demonstrated to reduce seizures significantly. Finally, syndrome of the trephined is particularly unique to decompressive procedures [51]. It is characterised by headaches, dizziness, mood changes or seizures as a result of a sunken flap after oedema has resolved. It usually improves after replacement of a bone flap.

## Cranioplasty

In patients who undergo full craniectomy, the replacement of a bone flap weeks to months later will be required. The duration between primary surgery and craniooplasty varies between 1 and 12 months during which time there is an increased risk of injury to the unprotected brain [52]. This will be either replacement of the original flap or a synthetic cranioplasty. The original flap can either be stored in a certified tissue bank or in the abdominal subcutaneous wall of the patient. Abdominal storage requires both additional surgery to store and remove the flap and also holds potential risks of resorption, rhabdomyolysis and infection. Nevertheless, it offers a rapid replacement of the bone flap within a few weeks [Zabaty et al. 2015]. Alternatively, a synthetic implant can be used. These can be generated from polyetheretherketone (PEEK), porous polyethylene, acrylic or titanium.

## Conclusions

The collective evidence of small case series within the literature suggests that large decompressive surgeries with duraplasty can be effective in paediatric patients with early signs of neurologic deterioration or herniation and in treating intracranial hypertension refractory to medical management. It is proposed that the reversal of such pathology may assist in improving outcomes in critically ill patients who have sustained a severe TBI. Although there is good evidence that DC controls ICP, the operation is associated with complications and the decision to undertake the procedure requires careful assessment and appropriate assessment of parents/guardians.
